# Allele-Specific, Age-Dependent and BMI-Associated DNA Methylation of Human *MCHR1*

**DOI:** 10.1371/journal.pone.0017711

**Published:** 2011-05-26

**Authors:** Stefanie Stepanow, Kathrin Reichwald, Klaus Huse, Ulrike Gausmann, Almut Nebel, Philip Rosenstiel, Martin Wabitsch, Pamela Fischer-Posovszky, Matthias Platzer

**Affiliations:** 1 Genome Analysis, Leibniz Institute for Age Research – Fritz Lipmann Institute, Jena, Germany; 2 Institute of Clinical Molecular Biology, Christian-Albrechts-University, Kiel, Germany; 3 Department of Pediatrics and Adolescent Medicine, University of Ulm, Ulm, Germany; Victor Chang Cardiac Research Institute, Australia

## Abstract

**Background:**

Melanin-concentrating hormone receptor 1 (MCHR1) plays a significant role in regulation of energy balance, food intake, physical activity and body weight in humans and rodents. Several association studies for human obesity showed contrary results concerning the SNPs rs133072 (G/A) and rs133073 (T/C), which localize to the first exon of *MCHR1*. The variations constitute two main haplotypes (GT, AC). Both SNPs affect CpG dinucleotides, whereby each haplotype contains a potential methylation site at one of the two SNP positions. In addition, 15 CpGs in close vicinity of these SNPs constitute a weak CpG island. Here, we studied whether DNA methylation in this sequence context may contribute to population- and age-specific effects of *MCHR1* alleles in obesity.

**Principal Findings:**

We analyzed DNA methylation of a 315 bp region of *MCHR1* encompassing rs133072 and rs133073 and the CpG island in blood samples of 49 individuals by bisulfite sequencing. The AC haplotype shows a significantly higher methylation level than the GT haplotype. This allele-specific methylation is age-dependent. In young individuals (20–30 years) the difference in DNA methylation between haplotypes is significant; whereas in individuals older than 60 years it is not detectable. Interestingly, the GT allele shows a decrease in methylation status with increasing BMI, whereas the methylation of the AC allele is not associated with this phenotype. Heterozygous lymphoblastoid cell lines show the same pattern of allele-specific DNA methylation. The cell line, which exhibits the highest difference in methylation levels between both haplotypes, also shows allele-specific transcription of *MCHR1*, which can be abolished by treatment with the DNA methylase inhibitor 5-aza-2′-deoxycytidine.

**Conclusions:**

We show that DNA methylation at *MCHR1* is allele-specific, age-dependent, BMI-associated and affects transcription. Conceivably, this epigenetic regulation contributes to the age- and/or population specific effects reported for *MCHR1* in several human obesity studies.

## Introduction

DNA methylation is an essential epigenetic modification of the genome, and is involved in many cellular processes like transcription, X chromosome inactivation, genomic imprinting and chromosome stability [1], [2]. In mammals, DNA methylation occurs mainly at the cytosine of CpG dinucleotides, which are unevenly distributed across the genome [3]–[5]. Generally, CpGs are depleted, possibly because of high mutability of the methylated cytosine to thymine [6]. However, some genomic regions show less depletion of CpGs. Such CpG islands frequently overlap with the transcriptional start sites (TSS) of genes [1], [7], [8]. DNA methylation around the TSS can repress gene expression in two ways, either directly by inhibition of binding of transcription factors or indirectly by recruiting methyl-CpG-binding proteins and associated repressive chromatin remodelling activities [1], [2]. In contrast, DNA methylation in the gene body is associated with elevated gene expression [9].

Different DNA methylation levels of alleles of a given gene within one cell have been observed in imprinted regions on a parent-of-origin basis [10], [11] and in X chromosome inactivation in females [12]. Moreover, allele-specific methylation (ASM) in autosomes, which is independent of parent-of-origin, was reported in humans [13]–[17]. Accordingly, about 10% of human genes may be affected by ASM, yet to date there are only few genes known to undergo ASM [15]. For example, only 12 loci showing ASM were identified in a recent genome-wide analysis [13]. Further, a recent methylation analysis of human chromosome 21 revealed two new loci, that undergo ASM and further confirmed one locus, which was previously identified [14], [16]. In a further, recent genome-wide study, 1.5% of the analyzed single nucleotide polymorphisms (SNPs) showed ASM, of which 90.3% appear to be in *cis* [17].

Allele-specific expression (ASE) is a widespread phenomenon in human cells [18], [19] and ASM likely contributes to it [13], [20]. Both aberrant ASE and DNA methylation are frequently associated with cancer and imprinting disorders (reviewed in [2], and references therein), but have also been reported for complex diseases like major psychosis [21]. In aging and/or tumor cells, global hypomethylation can lead to chromosomal instability, activation of transposable elements, loss of imprinting and expression of oncogenes. Local areas can gain methylation, for example CpG islands overlapping with promoters of tumor-suppressor genes, which can lead to a silencing of such genes as observed for *MLH1* and *BRCA1* [22]. Age-dependent effects on DNA methylation were also shown in a non-cancer-context [23]–[26]. These can be induced by either non-random mechanisms like responses to environmental changes or by stochastic errors in maintaining patterns of DNA methylation during cell proliferation [27]. Thus, age- and/or sequence-dependent changes in DNA methylation can have an impact on the etiology of diseases or phenotypic variability.

Melanin-concentrating hormone receptor 1 (MCHR1) plays a significant role in regulation of energy balance, food intake and body weight in humans and rodents [28]–[31]. To date, five human obesity association studies of SNPs in the *MCHR1* protein-coding region of exon 1 (rs133072: G/A, missense; rs133073: T/C, silent) were published and show inconsistent results or no association at all [32]–[36]. In adolescent German study groups (mean age ± standard deviation: 14±3 years and 25±4 years), association of the A allele of rs133072 and obesity was detected and supported by transmission disequilibrium. However, findings in other German and Danish, French and American study samples did not support the initial association. In the Danish sample (20±2 years) and in a second, epidemiological German sample (24–74 years) the frequency of the A allele of rs133072 was higher in non-obese *vs*. obese individuals, but not statistically significant [35]. In a French Caucasian group comprising obese children (<18 years, BMI>97^th^ percentile) and obese adults (BMI>40) the G allele of rs133072 was associated with obesity/BMI (*P* = 0.044) compared to adult controls [32]. Further, in Danish men (median age 47 and 49 years) a significant association of the rs133072 A allele with reduced abdominal obesity was found [34]. In contrast, two other groups did not find association of SNPs rs133072 and rs133073 with obesity in a population-based cohort of British Caucasians aged 40–65 years (mean BMI = 26) and a Finnish study group aged between 50–70 years, respectively [33], [36]. Further, the missense SNP rs133072 does not show obvious functional relevance *in vitro* [35].

These contrasting results suggest that SNP-dependent epigenetic variations may influence the association with obesity. The role of genotype-dependent DNA methylation in gene silencing/expression has previously been shown for the respiratory chain component *NDUFB6*, a gene associated with the risk of type 2 diabetes mellitus, in human skeletal muscle [37]. In the present study, we analyzed DNA methylation with respect to allelic status of SNPs rs133072 and rs133073 of the obesity candidate gene *MCHR1* [32]–[36].

SNPs rs133072 and rs133073 are located in the first exon of *MCHR1*. They are in tight linkage and form two major haplotypes, GT and AC [32], [35]; in these, one allele of either SNP constitutes a potential methylation site. We analyzed DNA isolated from blood cells of 49 individuals and found differential, haplotype-specific methylation levels. This ASM at *MCHR1* is age-dependent, which means the difference in methylation status between haplotypes is significant in young (20–30 y) but abolished in old individuals. Interestingly, the methylation status of the GT haplotype decreases with increasing BMI, whereas the AC haplotype shows no association in methylation status with BMI. In a *MCHR1* heterozygous lymphoblastoid cell line (LCL), which shows ASM, ASE could be abolished by treatment with the methylation inhibitor 5-aza-2′-deoxycytidine (AzadC).

## Results

### The *MCHR1* CpG island

**Figure 1:**
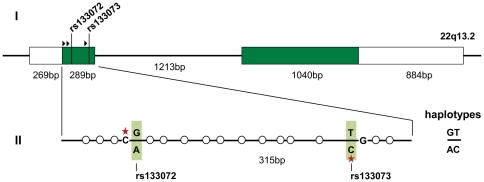
The genomic structure of *MCHR1* and the analyzed region. (I) Genomic structure of *MCHR1*. *MCHR1* is located on chromosome 22q13.2 and shown according to NM_005297. Boxes represent the two exons of *MCHR1*; white parts show untranslated regions (UTRs) and green parts show coding regions (CDS). Three potential translation start sites are indicated by black triangles. The SNPs rs133072 and rs133073 are located in the coding region of the first exon. (II) Detailed view of the analyzed region. White circles indicate CpGs. Alleles of SNPs rs133072 and rs133073 are highlighted in green. Red asterisks mark the potential methylation site created by one allele of either SNP. In our data set, only the two haplotypes GT and AC were observed.

The SNPs rs133072 (G/A) and rs133073 (T/C) form each a CpG, if allele G or C is present, respectively. Based on sequences of chimpanzee (CGSC 2.1/panTro2) and rhesus macaque (MGSC Merged 1.0/rheMac3), these alleles represent the ancestral state. In the vicinity of these SNPs there are 15 additional CpGs, which form a CpG island according to criteria put forward by Gardiner-Garden and Frommer [38]. In detail, the CpG island has a length of 220 bp, a G+C content of 66% and an observed *vs.* expected CpG ratio of 0.64. The CpG island is embedded in the coding portion of *MCHR1* exon 1 and is located about 300 bp downstream of the putative *MCHR1* TSS (human GRCh37/hg19 assembly: chr.22: 41,075,182, RefSeq: NM_005297) (Figure 1).

### DNA methylation at *MCHR1* is allele-specific

We initially genotyped the *MCHR1* SNPs rs133072 and rs133073 in 93 DNA samples of individuals aged between 21 and 78 years. All homozygous individuals showed only two haplotypes, GT and AC. In heterozygous individuals, all individuals who were heterozygous at rs133072 were also heterozygous at rs133073. For further analyses PCR products of 18 heterozygotes were cloned and sequenced, which allowed determination of haplotypes. Also here, only GT and AC haplotypes were found. Therefore and because of the previously reported tight linkage of these SNPs [32], [35], we assumed that only two haplotypes, GT and AC, occur in our data set. In the following, we will refer to GT and AC haplotypes as GT and AC alleles. The major allele is GT with a frequency of 66.3%, which is consistent with Hap Map data for CEU individuals (Utah residents with Northern and Western European ancestry from CEPH (Centre d'Etude du Polymorphisme Humain) collection) [39]. Genotype frequencies in our sample are 45.7% for homozygous GT alleles, 41.3% for heterozygous and 13.0% for homozygous AC individuals. The observed genotype frequencies in our data set are consistent with the Hardy-Weinberg-equilibrium (*P* = 0.468, Chi-square test). For subsequent methylation analyses only unrelated Caucasian individuals were used.

**Figure 2:**
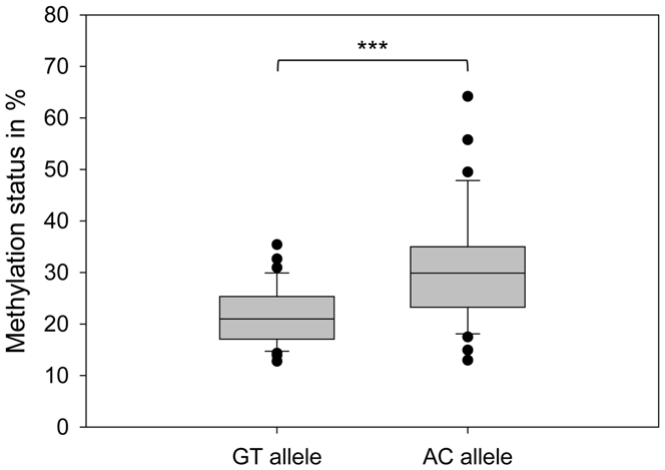
Allele-specific DNA methylation at *MCHR1*. The box plot shows average methylation levels of the GT and the AC allele observed in 49 individuals, comprised of 18 homozygous for GT, 13 homozygous for AC and 18 heterozygotes. The median methylation level for the GT allele is 20.9% and for the AC allele 29.9%. This difference is significant (***: *P*<0.001, *Mann-Whitney-test*). The methylation analysis was done by PCR on bisulfite treated DNA. PCR products were cloned and sequenced. We analyzed on average 41 clones per individual. The methylation level for each allele per individual was calculated by dividing the number of methylated sites in all clones harboring the respective allele by the number of possible methylation sites.

**Figure 3:**
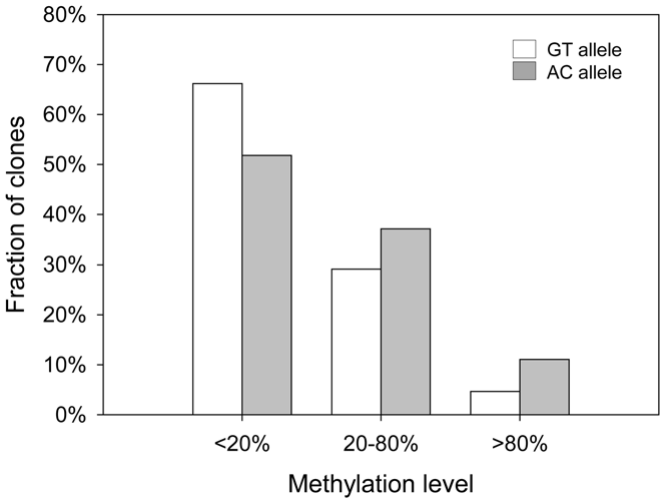
Fraction of clones with different methylation levels. The fraction of the GT allele bearing clones and that of AC allele containing clones are depicted in white and grey bars, respectively. The methylation level was calculated by dividing the number of methylated sites by the number of possible methylation sites in a single clone. As expected for a CpG island, the majority of clones are weakly methylated (<20%), but heterogeneously (20–70%) and highly methylated clones (>80%) were also observed. The AC allele showed a lower abundance in weakly methylated clones and a higher abundance in heterogeneously and highly methylated clones compared to the GT allele (*P*<0.001, Chi-square-test).

We next analyzed *MCHR1* methylation in blood of 49 individuals, including 18 individuals homozygous for GT, 13 individuals homozygous for AC and 18 heterozygotes after bisulfite treatment by cloning and sequencing. We analyzed on average 41 clones per individual. The average clone number for the GT allele is 29 (number of allele carrier: n_ind_ = 36) and for the AC allele 31 (n_ind_ = 31). The methylation intensity of the GT allele was significantly lower than that of the AC allele (median: 20.9% *vs.* 29.9%; *P*<0.001, *Mann-Whitney*-test; Figure 2). We also checked for methylation level differences according to gender and could not detect differences for both alleles (data not shown). As expected for a CpG island, the analyzed clones show a high abundance of unmethylated sequences (methyl-CpG/all CpG<20%), but heterogeneously (20–80%) and highly methylated clones (>80%) are also observed. Moreover, the AC allele shows a higher abundance in heterogeneously and highly methylated and fewer less methylated clones than the GT allele, which is significant (*P*<0.001, Chi-square-test) (Figure 3).

### DNA methylation at *MCHR1* is age-dependent

**Figure 4:**
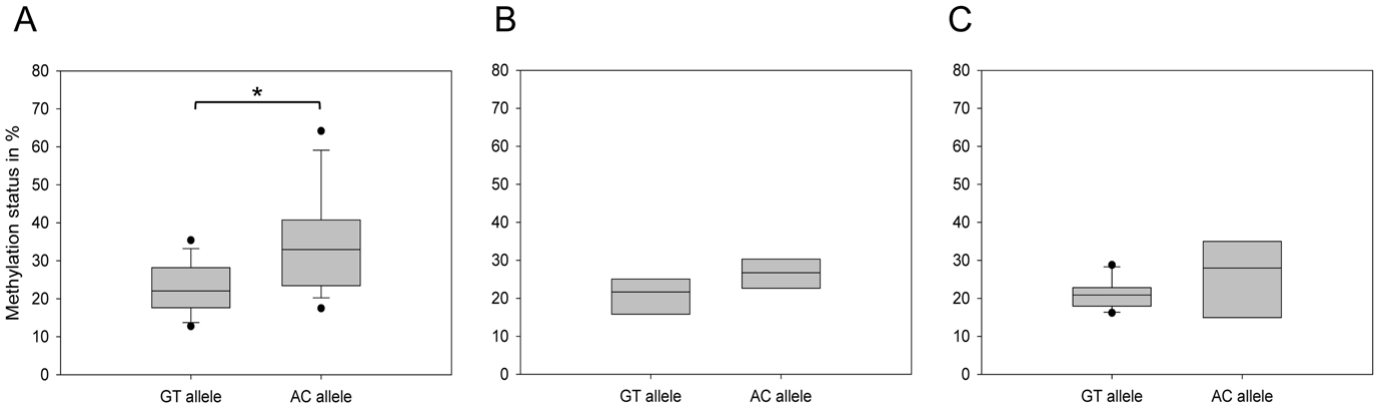
Age-dependent ASM at *MCHR1*. (A–C) The box plots show average methylation levels of the GT and the AC alleles of 45 individuals separated in three different age classes. *: *P*<0.05. **A:** ASM in the young group (20–30 years, n = 23; *P* = 0.003, *t*-test). The GT allele exhibited a mean methylation of 22.9±6.7%, while the AC allele was 34.2±12.7% methylated. **B:** ASM in the intermediate group (40–50 years, n = 10; *P* = 0.053, *t*-test). The GT allele shows a mean methylation of 20.7±5.1% and the AC allele is 26.5±4.4% methylated. **C:** Median methylation levels of GT and AC alleles in old individuals (>60 years, n = 12), which is 20.9% and 27.9%, respectively. This difference is not significant (*P* = 0.407, *Mann-Whitney-test*). **D:** Methylation levels of GT and AC alleles plotted against age (n = 49). The regression curves are depicted as a solid line for the GT allele methylation data and as a dashed line for the AC allele. The methylation status decreases with advancing age for both alleles, but this is not significant (GT: r = −0.059; *P* = 0.234; AC: r = −0.168; *P* = 0.144).

To test whether *MCHR1* methylation intensity varies over age, we further selected from the 49 individuals three age classes: young (20–30 years), intermediate (40–50 years) and old (>60 years), comprised of 23, 10 and 12 individuals, respectively. Genotypes of rs133072 in the age classes are distributed as follows: young (GG/GA/AA: 8/9/6), intermediate (4/3/3) and old (5/5/2). Again, in young individuals the methylation level of the GT allele was significantly lower compared to the AC allele (mean ± sd: 22.9±6.7% *vs.* 34.2±12.7%; *P* = 0.003, *t*-test; Figure 4A). In the intermediate group a less pronounced difference in methylation level of GT and AC alleles was observed (20.7±5.1% *vs.* 26.5±4.4%; *P* = 0.053; Figure 4B). In contrast, methylation levels of GT and AC alleles in old individuals did not differ significantly (median: 20.9% *vs.* 27.9%; *P* = 0.407, *Mann-Whitney*-test; Figure 4C). In conclusion, the ASM is significant in young in contrast to old individuals. A correlation analysis does not reveal a significant correlation of age and methylation status for both alleles (GT: r = −0.059; *P* = 0.234; AC: r = −0.168; *P* = 0.144, *Pearson correlation*), but show a decrease in methylation status with advancing age for both alleles, with a higher slope for the higher methylated AC allele. No gender difference was observed within the different age classes (data not shown).

### DNA methylation at *MCHR1* is BMI-associated

**Figure 5:**
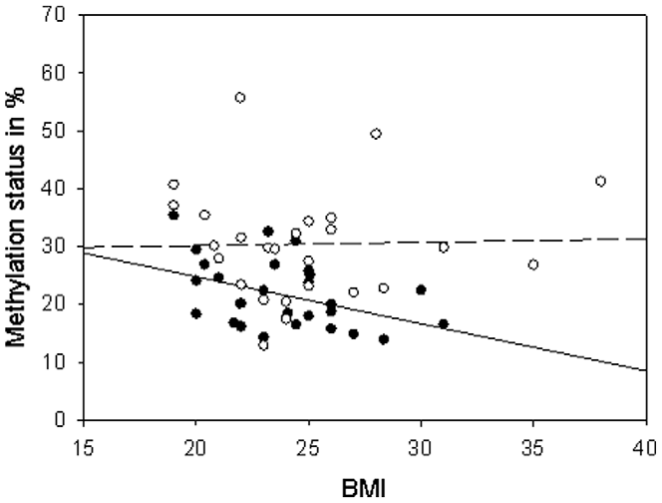
BMI-dependent DNA methylation levels at *MCHR1*. Allele specific methylation levels plotted against BMI of 39 individuals. The methylation status of the GT allele shows a significant negative correlation with BMI (r = −0.814, *P* = 0.024, *Pearson correlation*), whereas the methylation of the AC allele does not change with increasing BMI (r = 0.057, *P* = 0.897, *Pearson correlation*).

Furthermore, for 39 individuals with available BMI data (rs133072 genotypes GG/GA/AA: 13/16/10), we analyzed the methylation status *vs.* BMI. The methylation status of the GT allele (n_ind_ = 29) is negatively correlated with BMI (r = −0.814; *P* = 0.024, *Pearson correlation*), whereas for the AC allele (n_ind_ = 26) we did not detect a difference in methylation with respect to increasing BMI (r = 0.057; *P* = 0.897, *Pearson correlation*; Figure 5).

### ASM and ASE of *MCHR1* in LCLs

**Figure 6:**
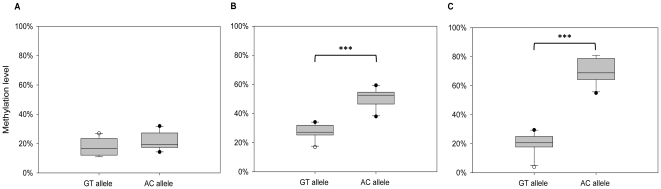
Allele-specific methylation levels of three LCLs. Mean methylation levels for GT and AC alleles were measured at ten time points of 63 passages of three heterozygous LCLs (for details see Figure S1). **A:** Mean methylation levels of both alleles of LCL GM12760. Mean methylation level was little at both alleles and does not differ (*P* = 0.230, *t*-test). The GT allele was on average 17.9±6.1% methylated, while the AC allele showed 21.3±5.8% methylation, which is not significantly different (*P* = 0.224, *t*-test). **B:** ASM of LCL GM12864. The GT allele is on average 27.5±5.2% methylated, whereas the AC allele showed a mean methylation level of 50.9±6.6%. This difference is significant (*P*<0.001, *t*-test). **C:** ASM of LCL C0913. The GT allele showed an average methylation of 20.2±7.3%, whereas the AC allele exhibited 70.2±8.5%. This difference is significant (*P*<0.001, *t*-test).

To examine whether there is a correlation between *MCHR1* methylation and mRNA expression, we studied three EBV transformed LCLs: GM12760, GM12864 and C0913, which are heterozygous at rs133072 and rs133073. At ten time points within 63 passages DNA methylation was stable in all three cell lines (Figure S1A–C). In GM12760 mean methylation intensity was little and did not differ significantly between alleles (GT: 17.9±6.1%, AC: 21.3±5.8%; *P* = 0.224, *t*-test; Figure 6A). GM12864 alleles were higher methylated and showed significant ASM (GT: 27.5±5.2%, AC: 50.9±6.6%; *P*<0.001; Figure 6B). LCL C0913 exhibited the most pronounced ASM (GT: 20.2±7.3%, AC: 70.2±8.5%; *P*<0.001; Figure 6C).

**Figure 7:**
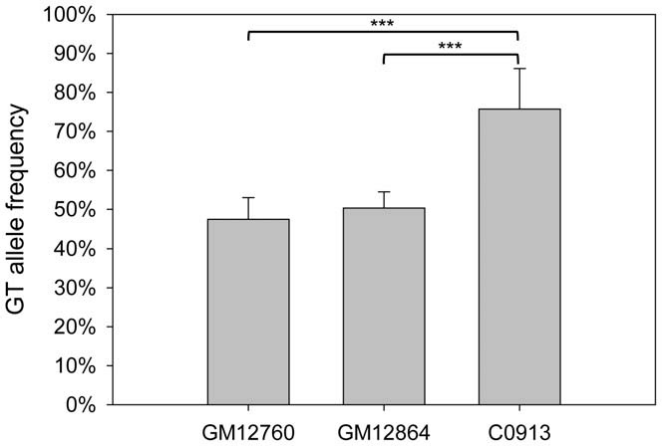
Allele-specific mRNA expression of LCLs. Mean frequency of the GT allele in cDNA is shown for three heterozygous LCLs GM12760, GM12864 and C0913 as measured by pyrosequencing at five time points, which were also analyzed for methylation level. GM12760 and GM12864 showed an equal transcription of both alleles, whereby GM12760 exhibited on average 47.4±5.6% of GT alleles and GM12864 50±4.2%. The LCL C0913, which exhibits the highest AC allele methylation level showed an average frequency of 75.7±10.4% of GT alleles. This preferential GT allele expression is significantly different from the equal transcription observed in GM12760 and GM12860 (*P*<0.05, ANOVA). Allele frequencies were confirmed by cloning and sequencing of a single passage of the LCL.

By pyrosequencing, we analyzed *MCHR1* ASE in these cell lines at five time points. Both GM12760 and GM12864 did not show allele-specific transcription (Figure 7). This was confirmed by cloning and subsequent sequencing of cDNA from a single passage, which allowed calling of the respective alleles (number of GT/AC clones: GM12760 = 74/82, GM12864 = 33/28). In contrast, in LCL C0913, which showed the highest difference in methylation intensity between GT and AC alleles, we observed allelic imbalance of *MCHR1* transcription. Mean frequency of transcripts representing the GT allele was 75.7±10.4% (Figure 7). This was confirmed by cloning and sequencing using cDNA from a single passage (GT/AC = 51/16, GT-fraction: 76%). The preferential GT allele expression of C0913 is significantly different from the equal expression of both alleles observed in GM12760 and GM12864 (*P*<0.05, ANOVA). To check if the observed allele-specific transcription is not due to a different number of allele copies in these LCLs, we measured allelic status in genomic DNA by pyrosequencing. All three LCLs have equal copies of both alleles (Figure S2).

**Figure 8:**
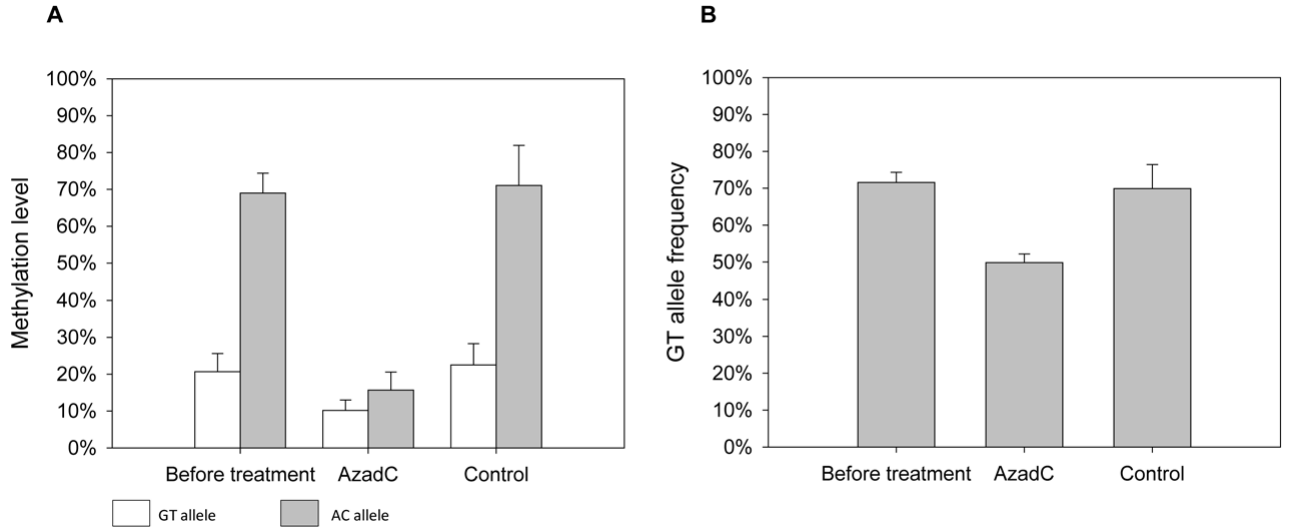
Treatment of LCL C0913 with AzadC. **A:** The ASM of LCL C0913 is shown before and following treatment with 5 µM AzadC. Mean methylation levels decreased from 20.7±4.9% to 10.2±2.8% for the GT allele and from 69±5.4% to 15.7±4.9% for the AC allele. Mean methylation level in the control cells did not change, that is, GT allele showed 22.5±5.8% and the AC allele 71.1±10.9%. **B:** ASE of LCL C0913 subjected to AzadC treatment. Allele frequencies were obtained by pyrosequencing of cDNA. Untreated cells showed allelic, that is more expression from the less methylated GT allele. By contrast, the allele frequency of the GT allele diminished from 71.6±2.8% to 49.9±2.3% following AzadC treatment. The control cells showed a GT allele frequency of 69.9±6.5%.

Next, C0913 cells were treated with the DNA methylase inhibitor AzadC. *MCHR1* methylation and transcription were analyzed after four days of treatment. Mean methylation decreased from 20.7±4.9% to 10.2±2.8% for GT alleles from 69.0±5.4% to 15.7±4.9% for AC alleles (Figure 8A), whereas the mean methylation in control cells did not change, that is, values were 22.5±5.8% for GT alleles and 71.1±10.9% for AC alleles, respectively. We measured changes in total expression of *MCHR1* in C0913 after AzadC treatment by qPCR and found a 645-fold increase of *MCHR1* transcripts compared to untreated cells (Figure S3). In parallel, the GT allele frequency in *MCHR1* transcripts dropped from 71.6±2.8% to 49.9±2.3% (Figure 8B). Control cells showed a GT allele frequency of 69.9±6.5%.

## Discussion

Here, we report the methylation analysis of a CpG island in the first exon of *MCHR1*, a gene involved in the control of energy metabolism and linked to obesity in rodents and humans [28]–[32], [34], [35], [40]. In blood, we found that *MCHR1* methylation is allele-specific, age-dependent, BMI-associated and affects gene expression. Generally, ASM is explained by an allele-specific affinity of DNA-binding proteins with downstream effects on DNA methylation and by direct effects of DNA sequence on propensity for methylation [13]. The analyzed region differs only in two positions (at SNPs rs133072 and rs133073) and the two major haplotypes show the same G+C and CpG content. Therefore we conclude that the observed ASM of *MCHR1* is due to sequence characteristics introduced by SNPs rs133072 and rs133073, but this does not exclude that linked variations outside of the analyzed region could be causative. However, the genes flanking *MCHR1* (*MKL1* and *SLC25A17*) did not show ASE in a study of lymphoblastoid cell lines [19]. Furthermore, there are no imprinted loci reported in the genomic context of *MCHR1* [41]. Remarkably, the observed ASM at *MCHR1* is age-dependent. The AC allele was significantly more methylated than the GT allele in individuals of young (20–30 years) in contrast to those of intermediate (40–50 years) and old (>60 years) age. Both alleles showed a decrease in methylation intensity with increasing age but with a smaller slope for the GT in comparison to the AC allele. It was previously shown that DNA methylation varies over age [23], [25], [42], [43]. An age-related loss of methylation can be explained by reduced fidelity of the maintenance methyltransferase DNMT1, whereas an age-related increase in methylation could potentially reflect the accumulation of stochastic methylation events [24]. In an Icelandic population sample it was shown that 29% of the individuals exhibit more than a 10% global methylation change (enriched for promoter regions) over time, whereby loss and gain in methylation intensity was observed. This was confirmed in a second study sample comprising individuals from a collection of Utah pedigrees [23]. Additionally, a familial clustering of global methylation changes over time was observed, which indicates a genetic mechanism underlying the methylation maintenance [23]. Although we currently cannot rule out that potential lineage-specific differences in methylation and changes in cell composition over age may contribute to the observed age-dependent methylation differences, the fact that we did not observe gender-specific effects despite significant differences in blood cell composition between males and females [57] argues against this possibility.

ASE is a widespread phenomenon in the human transcriptome [18], [44]. Because DNA methylation is correlated with gene silencing [45], ASM is suggested to contribute to ASE ([13]; reviewed in [20]). Accordingly, a pronounced ASM in the first exon of *MCHR1* in LCL C0913 is reflected in a skewed mRNA transcription rate: the highly methylated AC allele has a three times lower expression than the lowly methylated GT allele. Further, global suppression of DNA methylation by AzadC supplementation leads to an elevated total *MCHR1* mRNA expression and abolishes ASE. The analyzed *MCHR1* CpG island is located 300 bp downstream of the putative *MCHR1* TSS. Although the island is weak and not located in the promoter region our results suggest that DNA methylation of this *MCHR1* CpG island has an impact on gene expression.

Mchr1-deficient (*Mchr1* ^−/−^) mice have a significantly elevated energy expenditure and show hyperactivity and resistance to diet-induced obesity [31], [46]. Mchr1 antagonists inhibit food intake, reduced consumption of palatable food, and, after chronic administration to rats with diet-induced obesity, resulted in a decrease in body weight (reviewed in [29]). Thus, a lower level of Mchr1 protein leads to a protection against obesity in rodents. Here, we show that DNA methylation in the first exon of human *MCHR1* may mediate suppression of gene transcription and thus cause a reduction of orexigenic effects of receptor ligands. That implies that the association of *MCHR1* and human obesity may be mediated epigenetically. Previously, a significant association of the A allele of rs133072 with obesity in a German study group comprising mainly adolescents could not be confirmed in other German, Danish, French and American study samples of older age [35]. In contrast, two other studies revealed an association of either the G allele of rs133072 and obesity in a French study sample [32] or the A allele with reduced abdominal obesity in Danish men [34], with both study samples being comprised of adults. Our data obtained in blood of individuals aged between 21 and 78 years – a higher methylation level of *MCHR1* associated with the A allele of rs133072 – suggests a protective effect of the A allele [32], [34] rather than an association with obesity [35]. This is supported by our observation of a significant decrease in methylation of the GT allele with increasing BMI, highlighting these epitypes as a potential risk factor for obesity. Resulting increased expression levels of hypomethylated GT alleles may therefore have a positive effect on food intake and BMI at a particular age and under specific environmental conditions.

We analyzed DNA methylation and mRNA expression in blood and in blood-derived cell lines, in which *MCHR1* is expressed at low level. Since ASM may be tissue specific [16], [17], we cannot conclude that our results represent a general feature of *MCHR1* in human tissues. Especially, in functionally relevant tissues like hypothalamus and adipose tissue [40], [47], [48], mechanisms and time course of *MCHR1* expression may differ compared to blood. Therefore, further analyses of the impact of allele-specific and/or age-dependent epigenetic variations of *MCHR1* on human obesity shall include adipose tissue available after lipectomy.

## Materials and Methods

### Study samples

Blood was drawn from 33 healthy volunteers from Jena, Germany (21–77 years, 11 males). For a further 60 individuals (20–78 years, 30 males) DNA isolated from blood was obtained from the popgen biobank [49]. These samples were grouped in three age classes, including young: 20–30 years, intermediate: 40–50 years and old: >60 years. Each age class contained 20 individuals with an equal proportion of male and female donors. After approval by the ethics committee of the University Medical Center Jena or of the Medical Faculty of Kiel, respectively, all individuals gave informed written consent. For methylation analysis we selected 49 individuals (21–78 years; 22 males).

### Cell culture

The B-lymphocyte, EBV transformed cell lines (LCL) GM12760 and GM12864 and cell line C0913 were purchased from The Coriell Institute for Medical Research (Camden, NJ, USA) and ECACC (Wiltshire, UK), respectively. Cell lines GM12760 and GM12864 are from male donors of the CEPH project, which comprises donors from Utah residents with ancestry from western and northern Europe (HapMap project). The donor of C0913 is an UK Caucasian female of unknown age. Cell lines were cultured in RPMI 1640 with GIBCO GlutaMAX™ (Invitrogen, Karlsruhe, Germany) with 15% Fetal Bovine Serum “GOLD” (PAA Laboratories, Pasching, Austria) and 1.5% PenStrep (Roth, Karlsruhe, Germany) in 25 cm^3^ and 75 cm^3^ BD Falcon™ flasks (Becton Dickinson, Heidelberg, Germany) at 37°C and 5% CO_2_ in a total amount of 10 ml and 30 ml, respectively. Cells were grown to a density of 1×10^6^ cells/ml and split in a ratio of 1∶3.

### DNA

DNA isolation from blood and cell lines was performed using the DNeasy Blood & Tissue Kit from Qiagen (Hilden, Germany) according to the manufacturer's protocol.

### PCR and genotyping

For genotyping of *MCHR1* SNPs rs133072 and rs133073 a nested PCR approach was used. Primers used in the first PCR were: M_Gt.1F 5′-GGAGATCCCTTTCCTGATGG-3′ and M_Gt.1R 5′-CCATCGCACCAGTGAGAGGC-3′. First PCR was performed in a volume of 25 µl. Cycling conditions were: 96°C for 5 min, 30 cycles at 95°C for 1 min, 59°C for 30 s, 72°C for 1 min 30 s and a final elongation step at 72°C for 10 min. In the second PCR, primers M_Gt.2F 5′-TGCAGGCATTCAGAAGTGG-3′ and M_Gt.2R 5′-CAAAGGTCTCATCCTGCTC-3′ were used. The PCR was done in a volume of 25 µl; conditions were 95°C for 2 min, 30 cycles at 95°C for 1 min, 56°C for 30 s, 72°C for 1 min and a final elongation step at 72°C for 10 min. Genotyping was performed by sequencing using BigDye Terminator v3.1 Sequencing Standard Kit (Applied Biosystems, Foster City, USA) and primers M_Gt.2F and M_Gt.3R 5′-CCTCAGAGCAAAGCAGACC-3′. Sequencing reactions were electrophoresed on ABI 3730×l automated sequencers. Base calling was performed using phred [50], [51]. Trace files were inspected visually in gap4 [52].

### Methylation analysis

A minimum of 200 ng DNA was treated with sodium bisulfite to convert unmethylated cytosines to uracil using the Methylation Gold Kit (Zymo Research, Orange, CA, USA). Bisulfite specific PCR (BSP) was performed using at least 10 ng of bisulfite treated DNA in 25 µl for 95°C for 30 s, 35 cycles at 60°c for 30 s, 72°C for 25 s, 95°C for 30 s and a final elongation step at 72°C for 5 min. BSP products were resolved on 1.5% agarose gels, purified using the Double Pure Kit (Bio&Sell, Feucht, Germany) and eluted in 12 µl HPLC water. Amplicons were cloned into pCR2.1 (TOPO-TA-Kit, Invitrogen). Further, *E. coli* (OneShot® TOP10 chemically competent cells, Invitrogen) were transformed according to the manufacturer's protocol. Single clones were sequenced using M13 primers as described. The methylation intensity for each individual was calculated by dividing the number of methylated sites in all clones by the number of possible methylation sites.

For *MCHR1*, the BSP product is 315 bp (position in the human GRCh37/hg19 assembly: chr.22 41,075,440–41,075,755) and contains 15 CpGs and SNPs rs133072 and rs133073. Primers for BSP were: M_BSP.1F 5′-TGTTTAGGTGATGTTAGTGGGAGTT-3′, M_BSP.1R 5′-ACTCCCAATCAACTCACCTAC-3′.

### mRNA expression analysis

Total LCL RNA was isolated from 10^7^ cells with Qiagen RNeasy Mini Kit (Qiagen). Reverse transcription was done using Omniscript™ RT Kit (Qiagen). Prior to further analysis, *MCHR1* cDNA was amplified using primers M_Gt.1F and M_Gt.1R as described above. PCR was performed in a volume of 25 µl. Cycling conditions were: 96°C for 5 min, 30 cycles at 95°C for 1 min, 59°C for 30 s, 72°C for 1 min 30 s and a final elongation step at 72°C for 10 min. For pyrosequencing biotinylated PCR products were needed. For this purpose, we carried out eight PCRs with one biotinylated and one unlabeled primer (for details see Tables S1 and S2). PCR was performed in 25 µl for 95°C for 30 s, 35 cycles at 60°c for 30 s, 72°C for 25 s, 95°C for 30 s and a final elongation step at 72°C for 5 min.

Allele frequencies were determined by pyrosequencing according to manufacturer's protocol. Briefly, biotin-labeled PCR products were immobilized on Streptavidin Sepharose™ (GE Healthcare, Munich, Germany) by mixing 20 µl of PCR product with 6 µl streptavidin Sepharose™ suspension, 10 µl water, and 40 µl 1×binding buffer, followed by shaking at room temperature for at least 10 min. To remove unbiotinylated DNA strand, samples were sequentially washed with 70% ethanol and 0.5 M NaOH using the PyroMark Vacuum PrepTool (Biotage). Immobilized single stranded DNA was then washed with 1× washing buffer for 10 s, transferred to 40 µl 1×annealing buffer plus 4 µl target-specific sequencing primer (10 pmol/µl in water), and kept at 80°C for 10 min. After equilibration to room temperature, sequencing was performed using sequencing primers (Table S2) and the Pyro Gold Reagent Kit (Biotage) in the PSQ 96MA Pyrosequencing instrument according to the manufacturer's instructions.

### Treatment with 5-aza-2′-deoxycytidine

Lymphoblastoid cells were counted and set at an initial concentration of 1×10^5^ cells/ml in a total volume of 6 ml per well. A single dose of 5 µM of AzadC (Sigma-Aldrich, Munich, Germany) was added to one well, while three wells were used as untreated controls. Cells were harvested after 96 hours; incubation medium was not changed. The experiment was repeated once.

### qPCR

Real-time PCR was performed with the iCycler iQ detection system (Bio-Rad, Munich, Germany). PCR reactions were performed in 50 µl volume using GoScript® qPCR Master Mix (Promega, Mannheim, Germany) following manufactory's protocol. All reactions were performed in triplicates and negative controls were always included. The cycle threshold (Ct) values were normalized to the Ct value which represented the lowest expression level. Fold changes describe the difference in expression level between untreated and treated LCL C0913. Ct values of *MCHR1* were normalized to Ct values of the housekeeping gene *GAPDH* (glyceraldehyde-3-phosphate dehydrogenase). For amplification, primers qM.F 5′-CCAGGCTACGGAGGAAGAC-3′ and qM.R 5′-GAGGTGATCCTGCCGAAGT-3′ were used for *MCHR1* and qG.F 5′-AACAGCGACACCCACTCCTC-3′ and qG.R 5′-GGAGGGGAGATTCAGTGTGGT-3′ for *GAPDH*. PCR conditions were 95°C for 2 min, 45 cycles at 95°C for 20 s, 59°C for 30 s, 72°C for 20 s and 80°C for 15 s following by melting curve analysis with 95°C for 30 s and a 0.5°C ramp starting from 75°C to 100°C.

### Data analysis

To detect CpG islands 2 kb up-and downstream of the putative TSS, the program CpG island searcher was used [53]. Parameters were set to the following criteria: G+C content 60%, observedCpG/expectedCpG ratio of 0.600, and a minimum length of 200 bp.

Hardy-Weinberg disequilibrium was tested using Chi square (**χ** ^2^) test in EXCEL (Microsoft Corporation, Unterschleißheim, Germany).

For primer design on bisulfite treated DNA, we used the MethPrimer software (http://www.urogene.org/methprimer) [54]. Primers for genotyping and pyrosequencing were designed manually or using Primer3 version 0.4.0 (http://frodo.wi.mit.edu/primer3/), respectively.

DNA methylation was analyzed using BIQanalyzer [55]. Quality parameters were “sequence error” and “bisulfite conversion rate”, which were set at 90% and 100%, respectively. Alignment of clone sequences was done using Clustal X [56]. To examine allele-specific methylation in heterozygous samples, we performed visual analysis of sequences, assigned alleles and calculated mean methylation and standard deviation in EXCEL.

Linear regression and Pearson correlation analysis was done using SigmaPlot® (Systat software Inc., Erkrath, Germany). Statistical analysis was performed using the t-test for normally distributed data. If the Normality Test (Shapiro-Wilk-test) failed, the non-parametric Mann-Whitney-test was used. To compare absolute clone counts for three different methylation levels (<20%, 20–80%, >80%), we performed a Chi square test, which was done in SigmaPlot®. To test, if the differences in allele-specific gene expression between the three LCLs do not occur by chance, we performed Kruskal-Wallis One Way ANOVA (Analysis of Variance) using SigmaPlot®. A value of *P*<0.05 was considered as statistically significant.

## Supporting Information

Figure S1 **Allele-specific DNA methylation at** ***MCHR1*** **in three LCLs.** DNA methylation levels of GT and AC alleles at ten single passages in the three analyzed heterozygous LCLs: A: GM12760, B: GM12864, C: C0913. The passage numbers were counted when cells were split after thawing of the immortalized LCLs. White circles display the methylation level of the AC allele; black circles show methylation level of the GT allele.
(TIF)

Figure S2 **GT allele frequency in genomic DNA of LCLs.** GT allele frequencies of the three LCLs in genomic DNA were obtained by pyrosequencing. The measurements were performed in a similar approach as for expression analysis of three LCLs (see methods). We used three primer pairs, which did not span exon-exon-boundaries. Both SNPs rs133072 and rs133073 were analyzed in independent PCRs. GT allele frequencies in genomic DNA were on average 50.23%±1.63 for LCL GM12760, 50.47%±0.68 for LCL GM12864 and 50.67%±1.17 for LCL C0913.
(TIF)

Figure S3 **Fold changes in total expression of** ***MCHR1*** **following AzadC treatment.** To check if global suppression of DNA methylation leads to an elevated *MCHR1* expression, we measured fold changes in gene expression by quantitative real time PCR. Following AzadC treatment, total expression of *MCHR1* changed about 645-fold.
(TIF)

Table S1 **Pyrosequencing primer names and sequences.**
(DOC)

Table S2 **Pyrosequencing PCR.**
(DOC)
