# Supplementary figures and images for: Allele-Specific, Age-Dependent and BMI-Associated DNA Methylation of Human MCHR1
Source: PLoS One. 2011 May 26;6(5):e17711. doi: 10.1371/journal.pone.0017711 (PMC3102661; doi:10.1371/journal.pone.0017711)

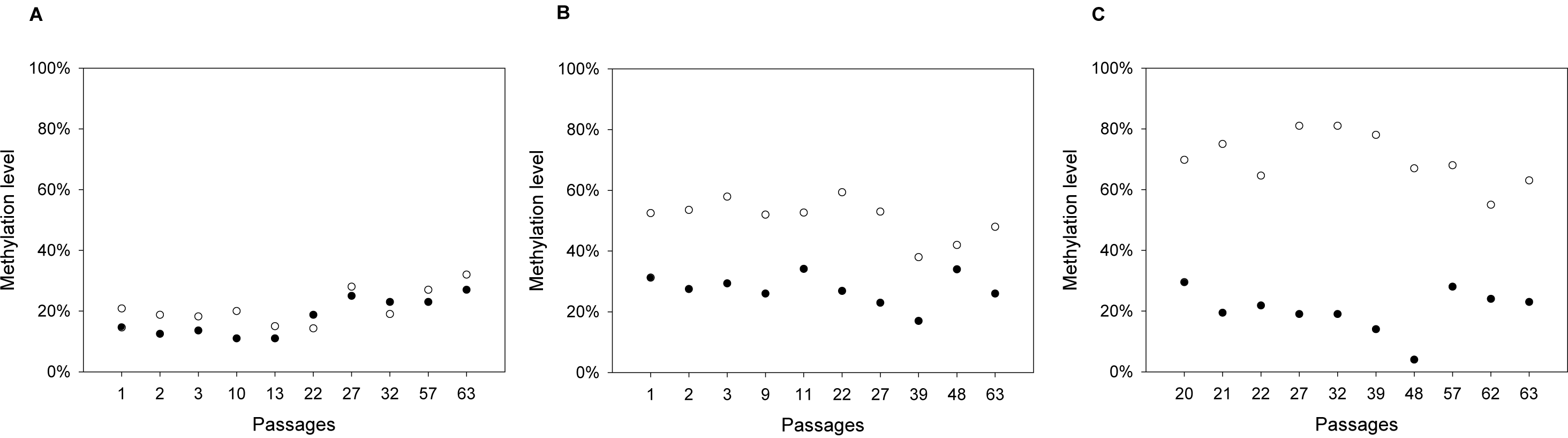

Supplement: Figure S1 — Allele-specific DNA methylation at MCHR1 in three LCLs. DNA methylation levels of GT and AC alleles at ten single passages in the three analyzed heterozygous LCLs: A: GM12760, B: GM12864, C: C0913. The passage numbers were counted when cells were split after thawing of the immortalized LCLs. White circles display the methylation level of the AC allele; black circles show methylation level of the GT allele. (TIF) [file pone.0017711.s001.tif]

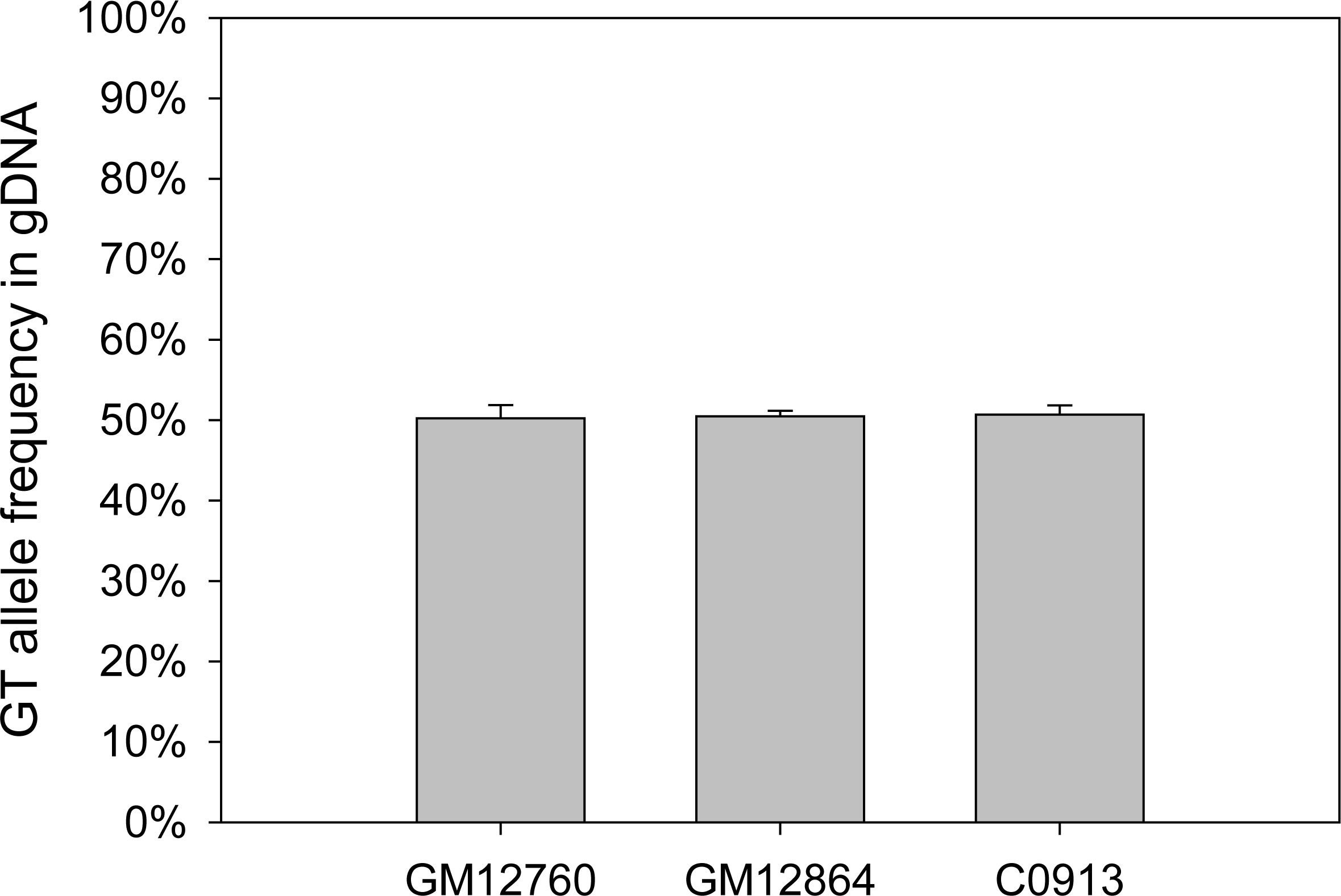

Supplement: Figure S2 — GT allele frequency in genomic DNA of LCLs. GT allele frequencies of the three LCLs in genomic DNA were obtained by pyrosequencing. The measurements were performed in a similar approach as for expression analysis of three LCLs (see methods). We used three primer pairs, which did not span exon-exon-boundaries. Both SNPs rs133072 and rs133073 were analyzed in independent PCRs. GT allele frequencies in genomic DNA were on average 50.23%±1.63 for LCL GM12760, 50.47%±0.68 for LCL GM12864 and 50.67%±1.17 for LCL C0913. (TIF) [file pone.0017711.s002.tif]

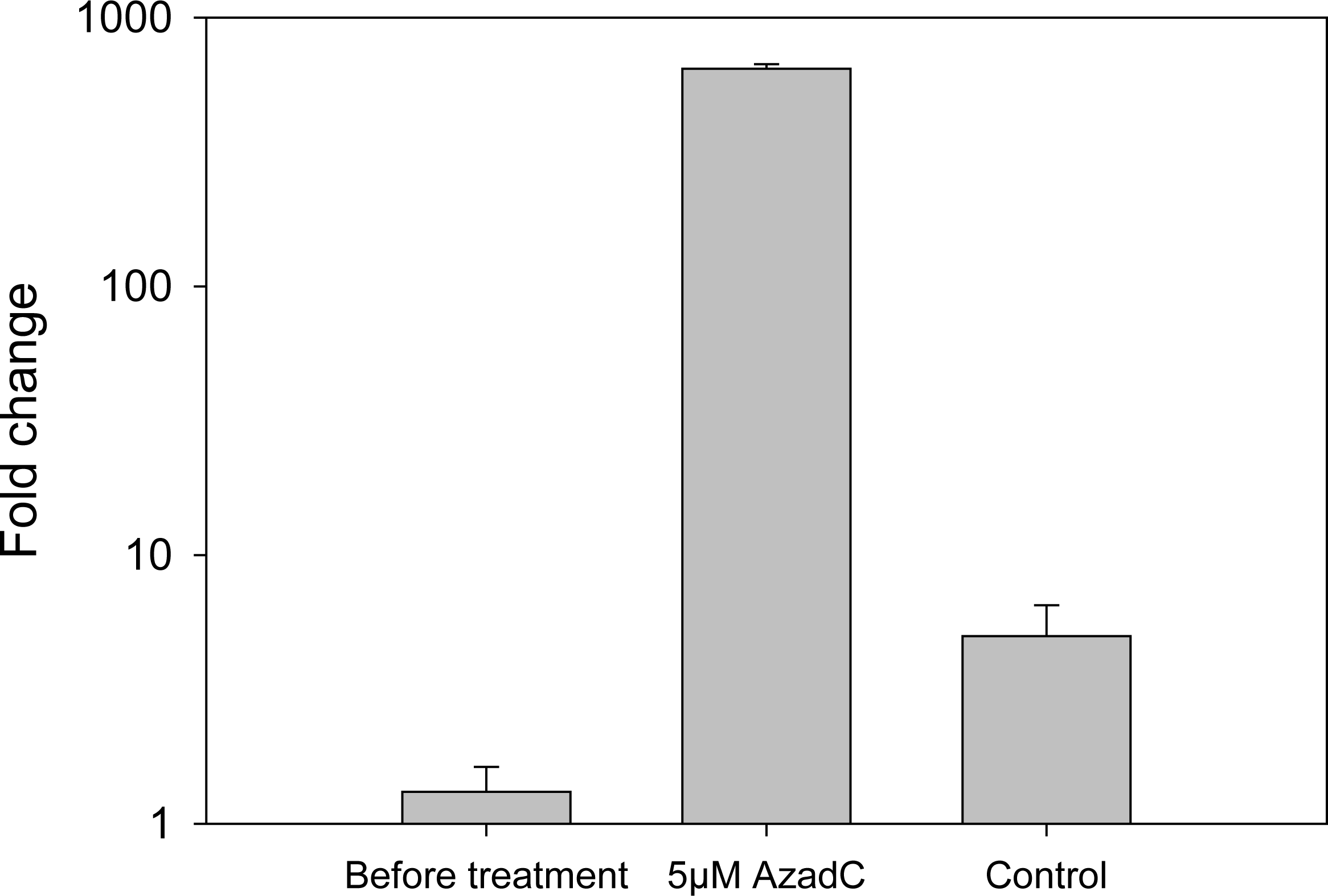

Supplement: Figure S3 — Fold changes in total expression of MCHR1 following AzadC treatment. To check if global suppression of DNA methylation leads to an elevated MCHR1 expression, we measured fold changes in gene expression by quantitative real time PCR. Following AzadC treatment, total expression of MCHR1 changed about 645-fold. (TIF) [file pone.0017711.s003.tif]
