# Supplementary material for: Allele-Specific, Age-Dependent and BMI-Associated DNA Methylation of Human MCHR1
Source: PLoS One. 2011 May 26;6(5):e17711. doi: 10.1371/journal.pone.0017711 (PMC3102661; doi:10.1371/journal.pone.0017711)
Supplement: Table S1 — Pyrosequencing primer names and sequences. (DOC) [file pone.0017711.s004.doc]

Table S1: Pyrosequencing primer names and sequences

| Primer name | Sequence |  |
| --- | --- | --- |
| bt_M_Gt.2F | 5’-TGCAGGCATTCAGAAGTGG-3’ | |
| bt_PSQ.1F | 5'-CCAGGCTACGGAGGAAGAC-3' | |
| bt_PSQ.1R | 5'-GAGGTGATCCTGCCGAAGT-3' | |
| PSQ.2R | 5'-AGCGCAAGCCCCGCAGT-3' | |
| PSQ.3R | 5'-CCATCAGAGGTGTTGCTGG-3' | |
| PSQ.4R | 5'-CATCAGAGGTGTTGCTGGC-3' | |
| PSQ.2F | 5'-GCTGCCCACTGGTCCCAA-3' | |
| PSQ.3F | 5'-TGCTGCCCACTGGTCCC-3' | |
| PSQ.1R | 5'-GAGGTGATCCTGCCGAAGT-3' | |
