# Supplementary material for: Allele-Specific, Age-Dependent and BMI-Associated DNA Methylation of Human MCHR1
Source: PLoS One. 2011 May 26;6(5):e17711. doi: 10.1371/journal.pone.0017711 (PMC3102661; doi:10.1371/journal.pone.0017711)
Supplement: Table S2 — Pyrosequencing PCR. (DOC) [file pone.0017711.s005.doc]

Table S2: Pyrosequencing PCR

| Product | PSQ-PCR primer | Product length | Sequencing primer |
| --- | --- | --- | --- |
| 1 | bt_M_Gt.2F + PSQ.2R | 198bp | PSQ.2R |
| 2 | bt_M_Gt.2F + PSQ.1R | 386bp | PSQ.2R |
| 3 | bt_PSQ.1F + PSQ.1R | 233bp | PSQ.3R |
| 4 | bt_PSQ.1F + PSQ.3R | 200bp | PSQ.3R |
| 5 | bt_PSQ.1F + PSQ.4R | 201bp | PSQ.4R |
| 6 | PSQ.2F + bt_PSQ.1R | 71bp | PSQ.2F |
| 7 | PSQ.3F + bt_PSQ.1R | 73bp | PSQ.3F |
| 8 | M_Gt.2F + bt_PSQ.1R | 386bp | PSQ.2f |
